# Predictive Modeling of MAFLD Based on Hsp90α and the Therapeutic Application of Teprenone in a Diet-Induced Mouse Model

**DOI:** 10.3389/fendo.2021.743202

**Published:** 2021-09-30

**Authors:** Yuan Xie, Lu Chen, Zhipeng Xu, Chen Li, Yangyue Ni, Min Hou, Lin Chen, Hao Chang, Yuxuan Yang, Huiquan Wang, Rongbo He, Rourou Chen, Li Qian, Yan Luo, Ying Zhang, Na Li, Yuxiao Zhu, Minjun Ji, Yu Liu

**Affiliations:** ^1^ Department of Endocrinology, Sir Run Run Hospital, Nanjing Medical University, Nanjing, China; ^2^ Department of Pathogen Biology, Jiangsu Province Key Laboratory of Modern Pathogen Biology, Center for Global Health, Nanjing Medical University, Nanjing, China

**Keywords:** MAFLD, NASH, steatohepatitis, Hsp90α, teprenone, geranylgeranylacetone

## Abstract

**Background and Aims:**

The heat shock protein (Hsp) 90α is induced by stress and regulates inflammation through multiple pathways. Elevated serum Hsp90α had been found in nonalcoholic steatohepatitis (NASH). Geranylgeranylacetone (GGA, also called teprenone) is a terpenoid derivative. It was reported to induce Hsp and alleviate insulin resistance. We aimed to evaluate the Hsp90α as a biomarker in predicting metabolic-associated fatty liver disease (MAFLD) and define the therapeutic effects of geranylgeranylacetone for the disease.

**Methods:**

A clinical study was conducted to analyze the elements associated with Hsp90α, and a predictive model of MAFLD was developed based on Hsp90α. The histopathological correlation between Hsp90α and MAFLD was investigated through a diet-induced mouse model. Furthermore, GGA was applied to the mouse model.

**Results:**

Serum Hsp90α was increased in patients with MAFLD. A positive linear relationship was found between age, glycosylated hemoglobin (HbA1c), MAFLD, and serum Hsp90α. Meanwhile, a negative linear relationship with body mass index (BMI) was found. A model using Hsp90α, BMI, HbA1c, and ALT was established for predicting MAFLD. The area under the receiver operating characteristic (ROC) curves was 0.94 (95% CI 0.909–0.971, *p* = 0.000). The sensitivity was 84.1%, and the specificity was 93.1%. *In vitro* experiments, GGA induced Hsp90α in steatosis cells. In the mice model, Hsp90α decreased in the GGA treatment group. Hepatic steatosis, inflammation, insulin resistance, and glucose intolerance were improved in the GGA-treated group. Serum Hsp90α was positively correlated with steatohepatitis activity according to hepatic histopathology.

**Conclusions:**

Serum Hsp90α was elevated in MAFLD, and a positive correlation between serum Hsp90α and the grade of activity of steatohepatitis was observed. The model using BMI, HbA1c, and alanine aminotransferase (ALT) had a good value to predict MAFLD. The findings also revealed the effectiveness of GGA in the treatment of MAFLD.

## Introduction

Metabolic-associated fatty liver disease (MAFLD), also known as nonalcoholic fatty liver disease (NAFLD), is closely associated with type 2 diabetes mellitus (T2DM), cardiovascular disease, and chronic kidney disease (CKD). Since the disease associated with metabolic dysfunction is common, “MAFLD” was suggested as a more appropriate overarching term (1). Today, new diagnostic criteria for MAFLD had been defined. As suggested in the consensus statement, the diagnosis of MAFLD is based on evidence of fat accumulation in the liver (hepatic steatosis) and one of the following three criteria: overweight/obesity, the presence of T2DM, or metabolic dysregulation (2). Compared with the NAFLD diagnostic criteria, the prevalence of MAFLD is less than NAFLD in patients with fatty liver disease. However, the definition of MAFLD is more practical for identifying patients with fatty liver disease with a high risk of metabolic comorbidities (3). For MAFLD, the consensus proposed that the assessment of the severity of MAFLD should be based on the grade of the activity and the stage of fibrosis in the liver (2). Hepatic steatosis [nonalcoholic fatty liver (NAFL)] and steatohepatitis [nonalcoholic steatohepatitis (NASH)] are the typical histological signs before cirrhosis (4). NASH is also an active form of NAFLD, requiring medical intervention once diagnosed since it is associated with cirrhosis. Unfortunately, there are limited biomarkers for detecting the activity of MAFLD in clinical settings. Alanine aminotransferase (ALT) is commonly used clinically to evaluate the presence of NASH. However, its level may be normal during disease progression (4, 5). Cytokeratin 18 (CK-18) fragment level is now the more recognized non-invasive biomarker for evaluating steatohepatitis, but the predicted value of CK-18 was reported as not satisfactory (6). Several other biomarkers have also been widely studied. These biomarkers represent the pathways involved in the development of steatohepatitis, including hepatocyte apoptosis, oxidative stress, and inflammation.

Metabolic syndrome (Mets) is a significant risk factor for NAFL/NASH. Meanwhile, NAFLD also increases the components of Mets (7). As reported, metabolic dysfunction is the critical feature in MAFLD (2, 8). It is well known that obesity, T2DM, and Mets are chronic inflammatory diseases (9). Chronic low-grade inflammation and activation of the immune system are involved in the pathogenesis of obesity-related insulin resistance and type 2 diabetes (10, 11). Hsp90α is an isoform of HSP90, induced and secreted under stress and inflammation. Similar to other heat shock proteins, Hsp90α also has unique cytoprotective functions, such as assisting in protein folding, facilitating cell signaling, and protecting cells from injury. Studies have also shown that Hsp90α is involved in regulating inflammation through multiple pathways (12–14). In some studies, Hsp90α was found to reflect the severity of inflammation (15, 16). It was reported that serum Hsp90α increased in NASH and correlated with the NAFLD activity score (17). Whether it could be used as a biomarker for detecting the activity of MAFLD is worth studying.

Many therapeutic agents for MAFLD have been investigated, targeting the regulation of energy metabolism, anti-inflammation, and antifibrosis. Furthermore, some of them are currently in clinical trials. However, since the duration of clinical trials is relatively short, the safety and efficacy of long-term drug use must be further clarified. Unfortunately, there are still no approved drugs for MAFLD. Lifestyle changes based on a healthy diet and regular exercise, and treatment of the concomitant components of the metabolic syndrome remain the primary modalities for treating the disease. GGA, also called teprenone, is a derivative of terpene and is widely used as an antipeptic ulcer agent in clinical practice. Teprenone is believed to promote the healing of acetic acid-induced chronic gastric ulcers that stimulate gastric mucus synthesis and secretion. Many studies have shown other effects of this medicine. It was found to inhibit neutrophil infiltration and enhance lipid peroxidation in ulcerated gastric tissues (18). Additionally, teprenone was reported to increase hepatic blood flow in rats with acute hepatic disorders due to carbon tetrachloride (CCL4) and improve inflammatory cell infiltration and fatty changes in the liver (19). It had even been reported to induce Hsp90α in gastric mucosa and sturgeon spermatozoa (20, 21). Additionally, GGA was found to reduce visceral fat and serum insulin in mice on a high-fat diet (22). Several studies have also reported its protective effect against cardiovascular disease, neuronal cell death, depression, and lung injury/fibrosis in animal models (23–26). However, the role in MAFLD is uncovered. In this study, we aimed to explore the relationship between Hsp90α and metabolic parameters in MAFLD. Furthermore, Hsp90α was evaluated as a biomarker for MAFLD. Additionally, we investigated the effect of teprenone in the treatment of MAFLD.

## Materials and Methods

### Clinical Study

The clinical study was conducted at the Affiliated Sir Run Run Hospital Nanjing Medical University from February 2021 to May 2021. A total of 113 patients with MAFLD and 72 healthy subjects were included. The inclusion criteria were as follows: (1) aged from 18 to 70 years old; (2) no underlying diseases that seriously affect cardio, pulmonary, or kidney function; and (3) fatty liver confirmed by ultrasound. The exclusion criteria were as follows: (1) presence of infectious or non-infectious inflammatory disorders; (2) use of drugs that cause lipid accumulation in the liver, including glucocorticoids, tamoxifen, amiodarone, or methotrexate; (3) other diseases that cause hepatic steatosis or combined hepatic disease other than the fatty liver disease; (4) history of surgery or other trauma within the past year; (5) acute myocardial or cerebral infarction within the past year; (6) pregnant or breastfeeding women; (7) cancer; (8) severe heart, brain, and kidney diseases (9); working in a high-temperature environment (10); drinking history (daily drinking >20 g); and (11) parasitic infection.

MAFLD was diagnosed according to the criteria proposed by an international expert panel (1, 2): evidence of hepatic steatosis was provided by liver biopsies, imaging, or blood biomarkers with one of the following conditions: (1) overweight or obesity (BMI ≥23 kg/m^2^), (2) type 2 diabetes (2021 American Diabetes Association), and (3) metabolic dysfunction.

Gender, age, height, and weight were recorded for all subjects. Body mass index (BMI) was calculated. All subjects underwent biochemical tests for ALT, aspartate aminotransferase (AST), blood urea nitrogen (BUN), creatinine (Cr), uric acid (UA), glycosylated hemoglobin (HbA1c), total cholesterol (TC), triglyceride (TG), high-density lipoprotein cholesterol (HDL-C) and low-density lipoprotein cholesterol (LDL-C).

Fasting blood was collected early in the morning. It was centrifuged at 1,000 rpm for 10 min after being stored at 4°C for 30 min. The upper serum was absorbed into a new tube and stored at −80°C for detecting Hsp90α by enzyme-linked immunosorbent assay (ELISA).

### Hepatocyte Steatosis Model

The FL83B murine hepatocyte cell line was derived in 1969 by Charity Waymouth at the Jackson Laboratory and was kindly provided by the Liver Transplantation Center of Jiangsu Province Hospital. Cells were cultured in a DMEM medium (with 4.5 g/L d-glucose, 319-005-CL, WisentBio, Nanjing, China) containing 10% FBS (Gibco, Waltham, MA, USA) and 1‰ penicillin-streptomycin (Hyclone, Logan, UT, USA). Cells were spread on a 12-well plate. A total of 200 μM of oleic acid (Sigma, St. Louis, MO, USA) and 100 μM of palmitic acid (Sigma) were added to each well. They were cultured for 24 h to establish the steatosis model. Cells were then stimulated with 1 μM of GGA (Selleck Chemicals, Houston, TXA, USA) for 24 h (the control group was stimulated with the same volume of solvent DMSO).

### Diet-Induced Metabolic-Associated Fatty Liver Disease Mouse Model

The male C57BL/6 mice were aged 4–5 weeks and purchased from the Experimental Animal Base of Nanjing Medical University. All experimental animals were raised in the Animal Experimental Centre of Nanjing Medical University (SPF, controlled environment of 12 h light/dark cycle, four per cage).

The mice were fed a Gubra Amylin NASH diet (NASH diet), resulting in MAFLD (weight gain, impaired glucose tolerance, insulin resistance, hepatic steatosis, and steatohepatitis) (27, 28). The control mice were fed a normal standard diet. The mice were randomly divided into two groups after being fed a regular diet for 1 week: (1) the CON group was fed a normal diet (10 kcal% fat, D12450J, Research Diets Inc.), (2) the NASH group was fed a NASH diet (40 kcal% fat (of these, 0% trans-fat and 46% saturated fatty acids by weight), 22% fructose, 10% sucrose, 2% cholesterol; D09100310, Research Diets Inc., Brunswick, NJ, USA).

The mice were then divided into four groups after 12 weeks. The CON-NS group had a normal diet + NS (normal saline). The CON-GGA group had a normal diet + GGA. The NASH-NS group had a Gubra Amylin NASH diet + NS. The NASH-GGA group had a Gubra Amylin NASH diet + GGA.

The body weight and food intake were recorded once a week until the end of the experiment. The blood was collected through orbital veins after overnight fasting. Then, mice were executed by severing the neck.

### GGA Treatment

A suspension was prepared by dissolving GGA (Eisai Co., Ltd., Suzhou, China) in NS. A total of 200 mg/kg/day of GGA was given to the treatment group for 12 weeks. The GGA suspension was mixed thoroughly before the intragastric administration to each mouse. Then, the control group was intragastric-administrated NS of the same volume.

### Intraperitoneal Glucose Tolerance Test and Insulin Tolerance Test

Intraperitoneal glucose tolerance test (*ip*. GTT) and insulin tolerance test (*ip*. ITT) were performed on the 24th week. *Ip*. GTT was performed after fasting overnight. Blood glucose was measured from the tail vein immediately at 0, 15, 30, 60, 90, and 120 min after being injected with 20% glucose at 2 g/kg. Food was removed for 6 h before ***i*** *p.* ITT. Mice were injected with 0.1 U/ml insulin at 0.75 U/kg. Blood glucose was measured at 0, 15, 30, 60, 90, and 120 min.

### Quantitative RT-PCR

The total RNA was extracted from the cells and mouse tissue using RNAiso Plus kits (TaKaRa Biotechnology Co. Ltd., Kusatsu, Japan). The RNA quantity was determined using a NanoDrop Ultramicro-Spectrophotometer (Thermo Fisher Scientific, Waltham, MA, USA). The reverse transcription of RNA was performed using the HiScript II Q RT SuperMix for qPCR (Vazyme Biotech Co., Ltd., Nanjing, China). Real-time PCR was performed using ChamQ Universal SYBR qPCR Master Mix (Vazyme Biotech Co., Ltd.) and detected by a LightCycler^®^ 96 Real-Time PCR System (Roche, Basel, Switzerland). The relative expressions of the genes were calculated using the ^2−ΔΔ^Ct method and were normalized to β-actin. The sequences of primers are available in the **Supplementary Material**.

### Total Protein Preparation

Operations were performed on ice. Cells and fresh tissues were washed with ice-cold PBS. Tissues were homogenized with an electric homogenizer. Proteins were extracted using a RIPA lysis buffer (Beyotime, Shanghai, China) with protease and phosphatase inhibitors (Thermo Fisher Scientific). The cell suspension and tissue homogenates were maintained at constant agitation for 30 min, and then an ultrasound was conducted with an ultrasonic crusher. The protein concentration was quantified using a BCA reagent (Thermo Fisher Scientific) per the manufacturer’s protocol. Last, a protein loading buffer was added to the protein solution. It was denatured in boiling water and stored at −20°C for later use.

### Western Blotting

An equal amount of protein from each sample was loaded into each lane for separation by SDS-PAGE and then transferred to the PVDF membranes (Merck Millipore, Billerica, MA, USA). After blocking them with 5% (w/v) skim milk powder dissolved in PBS containing Tween-20 (PBST) at room temperature for 2 h, the membranes were incubated at 4°C overnight with the primary antibodies. After washing them with PBST, the membranes were incubated at room temperature for 1 h with the horseradish peroxidase (HRP)-conjugated secondary antibodies. The density of each band was quantified by densitometric analysis with Image Lab 6.0 software. Antibody details are available in the **Supplementary Material**.

### Enzyme-Linked Immunosorbent Assay

ELISA was operated according to the product’s instructions. A total of 100 μl of the standard and sample were added to each well. The wells were covered with an adhesive strip and incubated for two hours at 37°C. Then, the liquid was removed from each well. Next, 100 μl of the biotin-antibody (1×) was added to each well. The wells were covered with a new adhesive strip and incubated for 1 h at 37°C. Each well was then aspirated and washed by wash buffer. After the last wash, any remaining wash buffer was removed by aspirating or decanting. Next, 100 μl of HRP-avidin (1×) was added to each well, covered with a new adhesive strip, and incubated for 1 h at 37°C. The aspiration/wash process was then repeated five times as described above. Next, 90 μl of TMB substrate was added to each well, and the wells were incubated for 15–30 min at 37°C and were protected from light. Then, 50 μl of Stop Solution was added to each well, and the plate was gently tapped to ensure thorough mixing. The optical density of each well was determined within 5 min using a microplate reader set to 450 nm. The ELISA kit details are available in the **Supplementary Material**.

### Oil Red O Staining

An Oil Red O Stain Kit (D027-1-1, Jiancheng Bioengineering Institute, Nanjing, China) was used. The oil red O solution was diluted at 5:4 (v/v). The cells were washed with precooled PBS twice and then fixed with 4% paraformaldehyde for 10 min. They were then stained by oil red for 8–10 min (avoiding light). Then, 60% isopropanol was used to differentiate for five seconds. The cells were then washed three times with distilled water. Hematoxylin was used to restain the nuclei. The cells were again washed with distilled water three times. The oil red O was at last extracted with isopropanol after microscope imaging. A spectrophotometer read the OD value at 510 nm for quantitative analysis.

### Histological Analysis and NAFLD Activity Score

Tissues were fixed in 4% paraformaldehyde and sectioned into 4 μm thickness after being paraffin-embedded. Multiple sections were prepared and stained with hematoxylin and eosin (H&E), and Masson trichrome for histological observations. Histologic findings were assessed in a blinded fashion by an independent pathologic expert. NAFLD activity score (NAS) was calculated as the sum of the scores for steatosis, lobular inflammation, and ballooning. Scoring was performed per the staging/grading system proposed by Kleiner et al. (29).

*Immunohistochemical staining* was performed as follows. The sections were deparaffinized and rehydrated, and they were placed into a citric acid (pH 6.0) antigen retrieval buffer for antigen retrieval in a microwave oven. A total of 3% hydrogen peroxide was used to block endogenous peroxidase activity. Then, the tissue was covered with 3% BSA and sealed at room temperature. A primary antibody was added to the sections and incubated overnight at 4°C and then incubated with an HRP-conjugated secondary antibody at room temperature. A newly prepared DAB color-developing solution was added. Hematoxylin stain solution was used to counterstain in the nucleus.

*Immunofluorescence staining* was performed according to the following procedure. The sections were deparaffinized and rehydrated, the slides were immersed in an EDTA antigen retrieval buffer (pH 8.0), and they were maintained at a sub-boiling temperature. A total of 3% hydrogen peroxide was used to block endogenous peroxidase activity, and then it was blocked with 3% BSA at room temperature. The sections were incubated with the first primary antibody overnight at 4°C. Then, they were covered with the secondary antibody and incubated at room temperature in a dark condition. Following this, the CY3-TSA solution was added and incubated in a dark condition. Then, the sections were incubated with the second primary antibody and the secondary antibody after microwave treatment at sub-boiling temperature. They were incubated with a FITC-TSA solution in a dark condition. Again, they were incubated with the third primary antibody and secondary antibody after microwave treatment at a sub-boiling temperature. The third secondary antibody was Cy5 conjugated. The DAPI solution was used for counterstain in the nucleus. Then, they were incubated with a spontaneous fluorescence quenching reagent. Finally, an antifade mounting medium coverslip was used. The images were detected and collected by a slice scanner. CaseViewer (v2.3) slice scanning software was used. The images were analyzed by ImageJ software. Four to five images were taken of each section for quantitative analysis. Antibody details are available in the **Supplementary Material**.

### Statistical Analysis

SPSS 23 software was used to analyze the data. For continuous variables, statistically significant differences between groups were determined using Student’s *t*-test, the Mann-Whitney *U* test, or a two-way ANOVA. The multiple comparisons test was conducted using LSD. Data were presented as mean ± SEM. Non-parametric tests between groups were performed using the Chi-square test and Kruskal-Wallis test. The Spearman or Pearson tests were used for correlation analysis between variables. False discovery rate (FDR) controlling procedures were used for multiple factors analysis. Binary logistic regression was used for multifactorial analysis, and the area under the ROC curve was used to assess the diagnostic efficacy. *p*-Values <0.05 were considered statistically significant; significant differences are presented as follows: *p < 0.05, **p < 0.01, ***p < 0.001.

## Results

### Characteristics of the Clinical Study

A total of 113 patients with MAFLD and 72 healthy subjects were included in the case-control study. BMI, ALT, AST, TC, TG, LDL-C, UA, and HbA1c were higher in MAFLD patients than in healthy subjects. In contrast, HDL-C was lower in MAFLD patients relative to healthy subjects (**Table 1**).

**Table 1:** Clinical features of the two groups.

Characteristic	Control (*n* = 72)	MAFLD (*n* = 113)	*p-*Value
Male/female	28/44	78/35	0.000*(*χ*²)
Age	40.5 (35, 49)	50 (41, 56)	0.000*(U)
History of smoking (%)	30.8	43.6	0.287 (*χ*²)
BMI (kg/m^2^)	22.55 (20.00, 24.90)	25.71 (23.95, 28.15)	0.000* (U)
BMI ≥23 kg/m^2^ (%)	45.8	86.7	0.000* (*χ*²)
HbA1c (%)	5.1 (5.0, 5.3)	6.8 (5.4, 8.8)	0.000*(U)
T2DM (%)	–	56.6	
IGT (%)	–	7.1	
SBP	120 (116, 125)	127 (117, 131)	0.162 (U)
DBP	74 (70, 82)	78 (72, 85)	0.097 (U)
HTN (%)	–	35.9	
ALT (U/L)	11.55 (9.0, 17.8)	22.00 (15.0, 36.1)	0.000* (U)
AST (U/L)	17 (15.0, 19.9)	20.10 (16.6, 25.0)	0.000* (U)
TC (µ/L)	4.38 (3.98, 4.90)	4.77 (4.08, 5.24)	0.022* (U)
TG (µmol/L)	1.03 (0.75, 1.34)	1.80 (1.31, 3.08)	0.000*(U)
HTG (%)	–	54	
HDL-C (µmol/L)	1.39 (1.18, 1.62)	1.00 (0.84, 1.25)	0.000* (U)
Hypo-HDL (%)	–	62.8	
LDL-C (µmol/L)	2.68 (2.20, 3.21)	2.90 (2.37, 3.61)	0.055 (U)
UA (µmol/L)	274.0 (220.8, 335.3)	334.7 (276.5, 385.7)	0.000* (U)
Hsp90α (ng/ml)	2.38 (1.21, 3.50)	4.65 (3.00, 8.28)	0.000* (U)

BMI, body mass index; HbA1c, glycosylated hemoglobin; T2DM, type 2 diabetes mellitus; IGT, impaired glucose tolerance; SBP, systolic blood pressure; DBP, diastolic blood pressure; HTN, hypertension; ALT, alanine aminotransferase; AST, aspartate aminotransferase; TC, total cholesterol; TG, triglyceride; HTG, hypertriglyceridemia; HDL-C, high-density lipoprotein cholesterol; hypo-HDL, hypo-HDL-cholesterolemia; LDL-C, low-density lipoprotein cholesterol; UA, uric acid; Hsp, heat shock protein.

Results are expressed as medians (Q25, Q75).

The significance was determined using the Chi-square test (χ²) or the Mann-Whitney U test (U). Bold values mean statistical significant. *p < 0.05.

### Elevation of Serum Hsp90α in MAFLD Was Related to Metabolic Factors

Serum Hsp90α in MAFLD patients was significantly higher than that in healthy subjects. Spearman’s correlation analysis showed that serum Hsp90α was positively correlated with age, sex, MAFLD, BMI, HbA1c, ALT, AST, and TG but negatively correlated with HDL-C. There were also differences between men (higher) and women (lower) (**Supplementary Table S1**). Multiple linear regression analysis showed a linear relationship between age, BMI, HbA1c, MAFLD, and serum Hsp90α. This suggests that these factors together influence serum Hsp90α. Interestingly, when the other variables were controlled, BMI negatively influenced serum Hsp90α (**Supplementary Table S2**).

### Establishment of a Predictive Model for MAFLD

**Figure 1:**
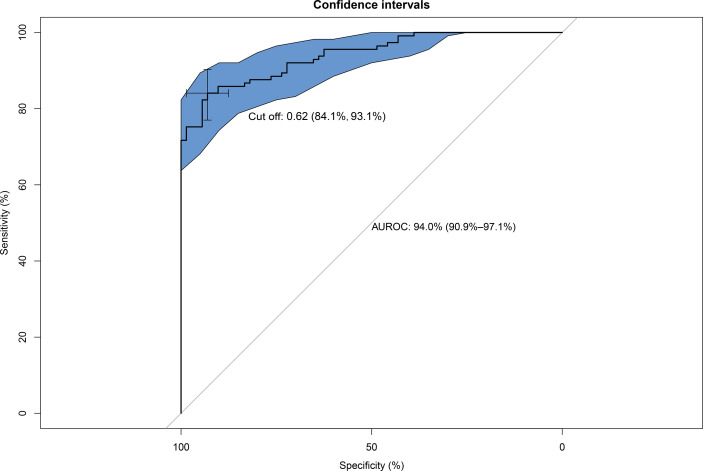
Area under the receiver operating characteristic (AUROC) curves of the model for predicting MAFLD using Hsp90α, BMI, HbA1c, and ALT. AUROC = 0.94 (95% CI, 0.909–0.971, *p* = 0.000), cut off 0.62, sensitivity 84.1%, and specificity 93.1%.

**Figure 2:**
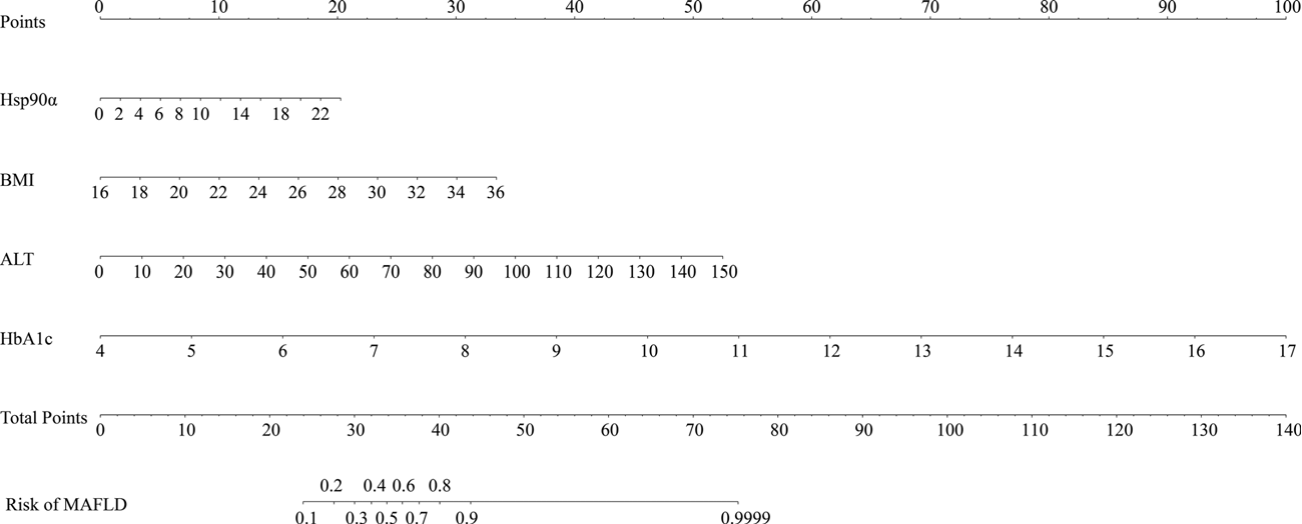
Nomogram for the model to predict MAFLD. The graph shows the risk of MAFLD concerning Hsp90α, BMI, HbA1c, and ALT, respectively. Each risk factor corresponds vertically to the corresponding “point” value. Furthermore, the “total points” is the sum of the points for all risk factors, vertically corresponding to the predicted “risk of MAFLD”.

Serum Hsp90α was used to predict MAFLD, but the area under the receiver operating characteristic (AUROC) curves was unsatisfactory (**Supplementary Figure S1**). The risk factors for MAFLD were analyzed by logistic regression (**Supplementary Table S3**). A predictive model for MAFLD was established by combining Hsp90α, BMI, HbA1c, and ALT (**Figure 1**). The equation was as follows: Risk = −20.283 + 1.206 × Hsp90α (ng/ml) + 1.449 × BMI (kg/m^2^) + 5.521 × HbA1c + 1.081 × ALT (U/L). The AUROC was 0.94 (95% CI 0.909–0.971, *p* = 0.000), with a sensitivity of 84.1% and specificity of 93.1%. A nomogram for the model is shown in **Figure 2**.

### Lipids Inhibited Hsp90α Expression in FL83b Cells, GGA Upregulated Hsp90α Expression, and Ameliorated Steatosis

**Figure 3:**
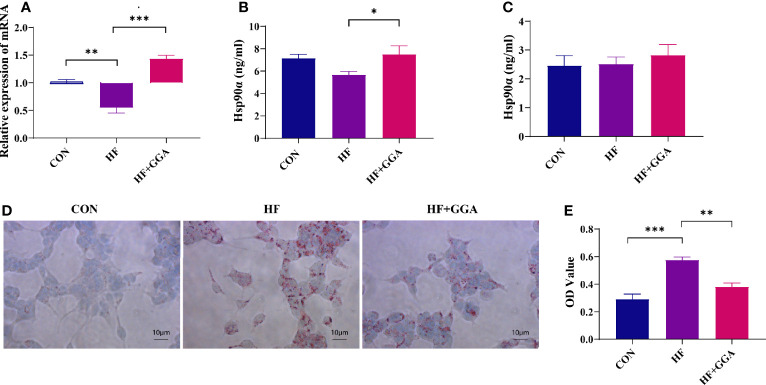
FL83b cell steatosis model. CON, control group; HF, steatosis group; HF+GGA, steatosis + 1 μM GGA group. **(A)** Expression of Hsp90α mRNA in FL83b cells. **(B)** Concentration of Hsp90α in FL83b cell homogenization tested by ELISA. **(C)** Hsp90α in supernatant derived from FL83b cells tested by ELISA. **(D)** Oil red O staining. **(E)** Quantitative analysis of oil red O staining. Data are presented as mean ± SEM. The significance among groups was determined using the two-way ANOVA with LSD’s multiple comparisons test.*p < 0.05, **p < 0.01, ***p < 0.001.

The expression of Hsp90α mRNA and protein was decreased in the steatosis group and increased in the GGA-stimulated group (**Figures 3A, B**). The concentration of Hsp90α in the supernatant did not change (**Figure 3C**). A marked reduction in the intracellular lipid droplets was observed in the GGA-stimulated group (**Figures 3D, E**).

### GGA Improved Mice Steatohepatitis

**Figure 4:**
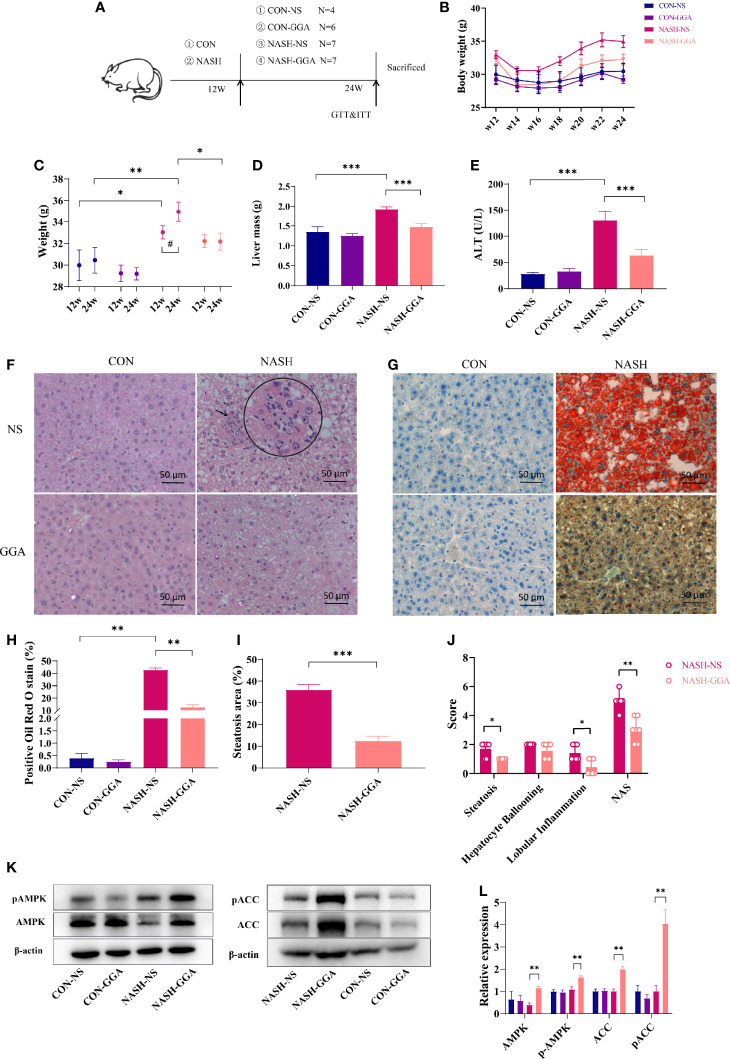
GGA improved steatosis and steatohepatitis in MAFLD mice. **(A)** Four to 5-week-old male C57BL/6 mice were fed a normal diet (CON group)/Gubra Amylin NASH diet (NASH group) for 12 weeks and then administered GGA/NS once a day for 12 weeks. The whole experiment lasted 24 weeks. **(B)** The dynamic changes in the body weights of mice from 12 to 24 weeks during the investigation. **(C)** Comparison of body weights at 12 and 24 weeks. *Comparison of body weight at 12w and 24w between groups; ^#^Weight changes from 12w to 24w. **(D)** Comparison of liver mass of mice in each group at 24 weeks. **(E)** Comparison of ALT levels of mice in each group at 24 weeks. **(F)** H&E staining of liver sections. The circle shows a zoom at the arrow. **(G)** Liver sections with oil red O staining. **(H)** Quantitative analysis of oil red O staining. **(I)** Comparison of hepatic steatosis area in the NASH-NS and NASH-GGA groups. **(J)** Comparison of NAS in the NASH-NS and NASH-GGA groups. **(K, L)** Expression of signaling proteins in each group. Data in **(C–E, H, I, L)** are presented as mean ± SEM. The significance among the groups was determined using the two-way ANOVA with LSD’s multiple comparisons test (^#^ in **(C)** was determined by paired samples *t*-test.). Those in **(J)** are presented as a mean with a range; Mann-Whitney *U* test. (*n* = 4 in CON-NS group, *n* = 6 in CON-GGA group, *n* = 7 in NASH-NS group, *n* = 7 in NASH-GGA group). *^,#^ *p *< 0.05.

The experiment lasted 24 weeks. Mice were fed a normal diet (CON group)/Gubra Amylin NASH diet (NASH group) for 12 weeks and then administered GGA/NS once a day for 12 weeks (**Figure 4A**). The NASH-NS group had a higher body weight (BW) than the CON-NS group at 12 and 24 weeks. The NASH-NS group gained more weight than the NASH-GGA group from 12 to 24 weeks, while the average body weight of the NASH-GGA group did not change much (**Figures 4B, C**). There was little variation in food intake between the groups (**Supplementary Figure S2**). The liver mass (**Figure 4D**) and ALT (**Figure 4E**) in the NASH-NS group were higher than the CON-NS and NASH-GGA groups, respectively. Steatosis, ballooning, and lobular inflammation were observed less seriously in the NASH-GGA group than in the NASH-NS group (**Figure 4F**). Oil red O stain also showed fewer lipid droplets contented in the NASH-GGA group (**Figures 4G, H**).

The actual area of hepatocyte steatosis was significantly lower in the NASH-GGA group than in the NASH-NS group (**Figure 4I**). Of the three parameters, the scores of hepatocyte steatosis and inflammatory were significantly lower in the NASH-GGA group than in the NASH-NS group. On the other hand, NAS in the NASH-GGA group was reduced significantly (**Figure 4J**).

The AMP-activated protein kinase (AMPK) was reported as an “energy regulator in cells”. It reduces lipogenesis through the phosphorylation of its downstream substrate acetyl-coenzyme A carboxylase (ACC). This is a process that inhibits the expression of lipid synthesis-related transcription factors (30, 31). The AMPK/ACC pathway is a crucial target for many agents that improve NAFLD (32, 33). Consistently, the levels of phosphorylated AMPK and ACC in the liver were enhanced in the NASH-GGA group (**Figures 4K, L**).

### GGA Improved Insulin Resistance and Glucose Intolerance in MAFLD Mice

**Figure 5:**
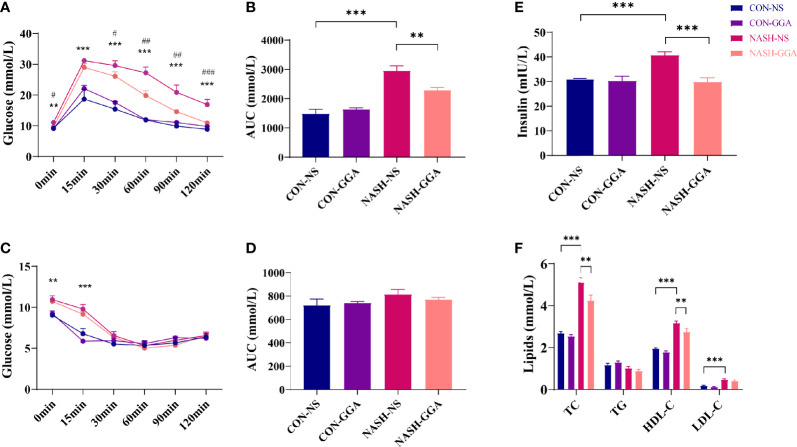
GGA improved insulin resistance and glucose intolerance in MAFLD mice. **(A)** Blood glucose at each time point during *ip.* GTT, *CON-NS *vs.* NASH-NS, ^#^NASH-NS *vs.* NASH-GGA. **(B)** Area under the curve of glucose for *ip.* GTT. **(C)** Blood glucose at each time point during *ip.* ITT, *CON-NS *vs.* NASH-NS, ^#^NASH-NS *vs.* NASH-GGA. **(D)** Area under the curve of glucose for *ip*. ITT. **(E)** Comparison of fasting serum insulin levels between the groups. **(F)** Comparison of lipid profile in each group. Data are presented as mean ± SEM. The significance among the groups was determined using the two-way ANOVA with LSD’s multiple comparisons test (*n* = 4 in CON-NS group, *n* = 6 in CON-GGA group, *n* = 7 in NASH-NS group, *n* = 7 in NASH-GGA group), *^,#^ *p *< 0.05, **^,##^ *p *< 0.01, ***^,###^ *p *< 0.001.

*Ip*. GTT and *ip*. ITT were performed on the 24th week. Both blood glucose during *ip*. GTT and the area under the curve (AUC) of glucose for *ip*. GTT were much higher in the NASH-NS group than the CON-NS group. In the NASH-GGA group, blood glucose was decreased significantly compared with in the NASH-NS group. The AUC of glucose for *ip*. GTT was also reduced (**Figures 5A, B**). The blood glucose at 0 and 15 min during *ip*. ITT in NASH-NS was higher than in the CON-NS group. Blood glucose in the NASH-GGA group was no different from the NASH-NS group. There were no significant differences in the AUC of glucose for *ip*. ITT between groups (**Figures 5C, D**).

We also tested the fasting serum insulin and lipids. The result showed increased serum insulin in the NASH-NS group and a decrease in the NASH-GGA group (**Figure 5E**). On the other hand, lipids (except TG) in the NASH-NS group were markedly increased. In the NASH-GGA group, while the TC and HDL-C decreased, they were still much higher than in the CON-NS group (**Figure 5F**).

### The Level of Serum Hsp90α Was Relevant to the Activity of MAFLD

**Figure 6:**
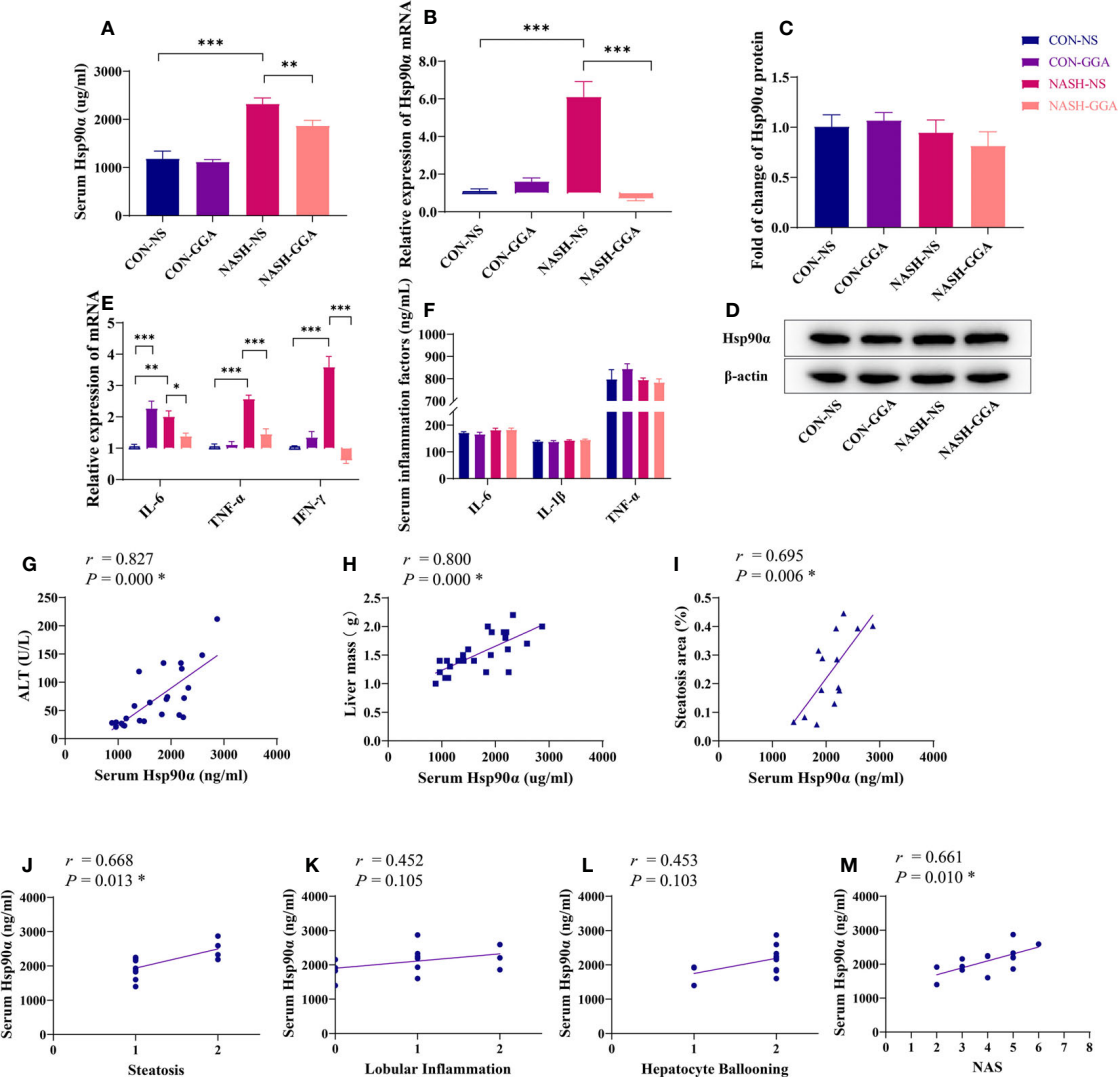
Level of serum Hsp90α was related to the activity of MAFLD. **(A)** The concentration of serum Hsp90α in each group. **(B)** Expression of hepatic Hsp90α mRNA in each group. **(C, D)** Expression of hepatic Hsp90 protein in each group. **(E)** Expression of proinflammatory cytokine mRNA in the liver. **(F)** Comparison of proinflammatory cytokines in serum. **(G–I)** Pearson’s correlation analysis between serum Hsp90α and ALT and the liver mass and steatosis area. **(J–M)** Spearman’s correlation analysis between serum Hsp90α and NAS. *p < 0.05 (FDR). Data in **(A–F)** are presented as mean ± SEM. The significance among the groups was determined using the two-way ANOVA with LSD’s multiple comparisons test (*n* = 4 in CON-NS group, n = 6 in CON-GGA group, *n* = 7 in NASH-NS group, *n* = 7 in NASH-GGA group).*p < 0.05, **p < 0.01, ***p < 0.001.

The expression of Hsp90α in the serum (**Figure 6A**) and the liver (**Figure 6B**) was significantly higher in the NASH-NS group than in the CON-NS group and lowered in the NASH-GGA group compared with the NASH-NS group. There were no significant differences in the Hsp90α protein content in the liver between the groups as determined by Western blotting (**Figures 6C, D**).

The NASH-NS group showed an increased expression of interleukin 6 (IL-6), tumor necrosis factor α (TNF-α), and interferon γ (IFN-γ) in the liver compared with the CON-NS group. Meanwhile, the NASH-GGA group showed reduced expression compared with the NASH-NS group. Curiously, IL-6 was also increased in the CON-GGA group (**Figure 6E**). However, there were no significant changes in the serum concentrations of IL-6, IL-1β, and TNF-α (**Figure 6F**).

Serum Hsp90α level was positively correlated with ALT (*r* = 0.827, *p* = 0.000), liver mass (*r* = 0.800, *p* = 0.000), the area of hepatic steatosis (*r* = 0.695, *p* = 0.006), the score of steatosis grade in the NAS (*r* = 0.668, *p* = 0.013), and the NAS (*r* = 0.661, *p* = 0.010) (**Figures 6G–M**).

### Increased Nuclear Transporting of Hsp90α Was Observed in MAFLD Mice

**Figure 7:**
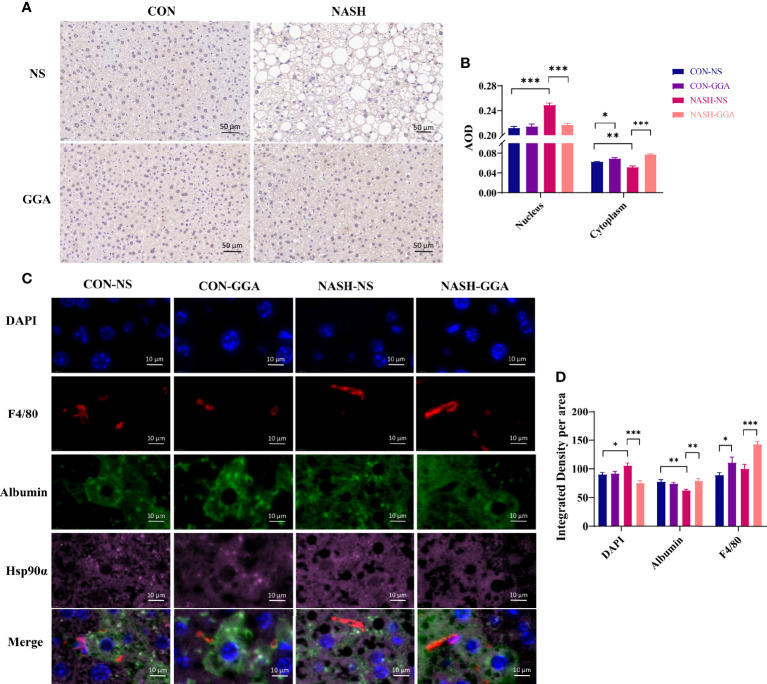
Increased nuclear transporting of Hsp90α in MAFLD mice. **(A)** Immunohistochemical staining of the liver section with Hsp90α. **(B)** Semiquantitative analysis of the expression of Hsp90α in the nucleus and cytoplasm in the immunohistochemical staining. **(C)** Immunofluorescence staining to investigate the locating of Hsp90α. DAPI (blue) marked the nucleus, F4/80 (red) marked Kupffer cells, albumin (green) marked hepatocytes. **(D)** Colocation analysis of Hsp90α and albumin, DAPI, F4/80 in immunofluorescence staining. Data are presented as mean ± SEM. The significance among groups was determined using the two-way ANOVA with LSD’s multiple comparisons test (*n* = 4 in CON-NS group, *n* = 6 in CON-GGA group, *n* = 7 in NASH-NS group, *n* = 7 in NASH-GGA group). *p < 0.05, **p < 0.01, ***p < 0.001.

There were significant changes in serum Hsp90α and liver Hsp90α mRNA expression in MAFLD mice in the above experiments but not in the Hsp90α protein. Studies reported that Hsp90α transports between cytoplasm and nucleus, and this transporting is enhanced by stress (34). Therefore, we performed immunohistochemical and immunofluorescence staining to evaluate the location of Hsp90α in the liver. The immunohistochemical staining showed less cytoplasmic but more nuclear Hsp90α expressed in the NASH-NS group (**Figures 7A, B**). Furthermore, the immunofluorescence staining analysis showed that the colocalization of Hsp90α and albumin was decreased in the NASH-NS group but increased in the NASH-GGA group. Conversely, the colocalization of Hsp90α and DAPI was raised in the NASH-NS group but fell in the NASH-GGA group (**Figures 7C, D**). Interestingly, increased Hsp90α in Kupffer cells was found both in the CON-GGA and the NASH-GGA group by analyzing the colocalization of Hsp90α and F4/80 (**Figure 7D**).

## Discussion

T2DM, obesity, and metabolic dysfunction are components of MAFLD. As wildly known, T2DM and fatty liver are very closely related. A total of 75% of T2DM has a combined fatty liver. Furthermore, compared with non-T2DM with fatty liver, patients with T2DM combined with fatty liver have an increased prevalence of steatohepatitis and advanced fibrosis, regardless of the ALT abnormality (35). Obesity is also closely associated with fatty liver, especially steatohepatitis, steatohepatitis-associated cirrhosis, and liver cancer. In the Chinese population, the prevalence of NAFLD in obesity is 70%–80% (36). For Italians, the prevalence of NAFLD was 75.8% in obesity and 16.4% in normal weight, with a relative risk of 4.6 times higher in obesity (37). Obese individuals have more severe liver inflammation and fibrosis than non-obese individuals. A study confirmed that obese individuals had higher NAFLD activity scores (NAS), with higher rates of hepatocyte ballooning formation, more significant inflammation, and more severe fibrosis in the liver compared with non-obese individuals (38). Insulin resistance is a common feature of the fatty liver, and it is also one of the causes of fatty liver (39). Insulin resistance reduces glucose utilization in nonhepatic tissues (including adipose tissue and muscle) and affects triglyceride metabolism in adipose tissue (40). About 60% of the fatty acids in the liver come from triglyceride lipolysis in adipose tissue (41). Obesity and insulin resistance cause the dysregulation of triglyceride lipolysis in adipose tissue, resulting in the excessive release of fatty acids and increase the lipid load on the liver, and insulin resistance is further aggravated (42). The excess fatty acids in hepatocytes are then converted into lipotoxic products through bypass pathways, leading to oxidative stress, injury, immune activation, and inflammation in the liver (43, 44).

In the current clinical study, BMI was found to have a negative effect on serum Hsp90α, suggesting that lipids may have an inhibitory effect on Hsp90α expression. This negative relationship of lipid accumulation and Hsp90α expression was further confirmed by the *in vitro* experiment results, where decreased Hsp90α mRNA and protein were observed in a steatosis cell model. Diametrically opposed to the *in vitro* experiments, the up-regulation of hepatic Hsp90α was observed in MAFLD mice. This might result in stress and inflammation, which were absent in the *in vitro* experiment. It has been reported that Hsp90α is usually induced under the condition of stress (45). In general, Hsp90α is not secreted unless the cellular environment changes (46). Stress states, such as reactive oxygen species (ROS), heat, hypoxia, radiation, injury, and cytokines, might induce the expression and secretion of Hsp90α (47). It has been reported that Hsp90α induces inflammation by activating the NF-κB and STAT3 pathways. The expression of Hsp90α is also induced by proinflammatory cytokines, including IL-6, IL-8, and NF-κB (14, 48). Costimulated pancreatic islet beta cells by TNF-α, IL-1β, and IFN-γ resulted in a large amount of Hsp90α secretion (16). The release of proinflammatory factors usually accompanies obesity, T2DM, and MAFLD. In a subgroup analysis, serum Hsp90α increased insignificantly in patients who both have MAFLD and T2DM (**Supplementary Figure S3**). The clinical study results suggested that elevated serum Hsp90α might be the result of metabolic abnormalities in the body. Alternatively, it might be a “mediator” implicated in the interaction among T2DM, overweight/obesity, hepatic steatosis, and steatohepatitis.

Different from Hsp90β, which is consistently expressed in tissues, Hsp90α is induced in response to stress. Though they are highly identical in amino acid sequences, with approximately 86% sequence homology, they differ significantly in the nucleotide. Their functions are also quite different (45, 49). Consistent with our studies, the expression of Hsp90β mRNA was quite different from Hsp90α, especially in the animal models (**Supplementary Figures S4**–**6**). In the study by Zheng et al., they found that overexpressed Hsp90β protein in a high-fat diet (HFD) induced an obese mouse model and the inhibition of Hsp90β improved lipid disorders in HFD mice. In another study on HFD mice, Hsp90α was increased and improved hepatic lipid metabolism in the treatment group (50) (**Supplementary Figure S4**). These two studies suggest entirely different functions of Hsp90α and Hsp90β. However, the hepatic protein of Hsp90α exhibited little change in metabolic disorders, consistent with the results of Zheng et al. (51). Interestingly, in the current study, immunofluorescence and immunohistochemical analysis revealed a change in the subcellular localization in the mice model. Hsp90α was increased in the nucleus and decreased in the cytoplasm of hepatocytes. It is reported that Hsp90α usually transports proteins between the cytoplasm and nucleus. Typically, 5%–10% of Hsp90α is in the nucleus. The transfer of Hsp90α to the nucleus increases with increasing stress (34, 52). Hsp90α binds to DNA, RNA, and histones in the nucleus, stabilizing the DNA structure and contributing to the synthesis and processing of RNA (34). Additionally, Hsp90α interacts with many transcription factors (TFs), including zinc finger proteins (ZFPs), helix-loop-helix (HLH) proteins, MyoD1, E12, hypoxia-inducible factor-1α (HIF-1α), and heat shock factor 1 (HSF-1) (34).

The analysis of hepatic histopathology revealed marked improvement of steatohepatitis when mice were treated with GGA. The alleviation of steatohepatitis was also reflected in other indicators, such as ALT, liver mass, and the expression of proinflammatory cytokines in the liver. These findings indicate that the change in Hsp90α corresponded to improved MAFLD. This study also revealed a histopathological correlation between serum Hsp90α and the activity of steatohepatitis according to the NAS. One study compared serum Hsp90α between NAFLD patients with non-steatohepatitis and steatohepatitis. It was found that Hsp90α was elevated in patients with steatohepatitis, and the serum level of Hsp90α was positively associated with the grade of activity, as measured by the NAS, steatosis grade, lobular inflammation grade, and hepatocyte ballooning grade (17). It suggests that Hsp90α might serve as a biomarker for predicting the activity of MAFLD. However, when Hsp90α was used to predict MAFLD in the current clinical study, both the sensitivity and specificity were not as desirable as expected. The development of MAFLD is also influenced by other factors, especially the combination of obesity and diabetes. Thus, a model was further established by combining Hsp90α, BMI, HbA1c, and ALT. Better results were obtained for this model.

Hsp90α is not only a serum biomarker but also an essential intracellular chaperone for many proteins. It has been reported that Hsp90 binds to both the γ and α subunits of AMPK with high affinity, regulating the stability of AMPK and promoting its activation. Therefore, it regulates the phosphorylation of downstream ACC to regulate the cellular fatty acid metabolism (53). Knocking down Hsp90 or using Hsp90 inhibitors can decrease the phosphorylation levels of AMPK and ACC (53). In the NASH-NS group, the reduction of Hsp90α in the cytoplasm may result in the transfer of Hsp90α to the nucleus or its secretion extracellularly. A deficiency of Hsp90α might influence client proteins in the cytoplasm to maintain their functions. In keratinized cells, Hsp90α promotes cell proliferation and facilitates wound healing through AKT activation; a deficiency of Hsp90α would affect wound healing (54, 55). In the retina, the deficiency of Hsp90α leads to retinitis pigmentosa (56). In this study, the upregulation of phosphorylation on AMPK/ACC was also found in the NASH-GGA group accompanied by an increase of cytoplasmic Hsp90α. However, whether this was related to Hsp90α needs further investigation.

In previous studies, two proteins purified from *Schistosoma japonicum* eggs were shown to have beneficial effects on carbohydrate and lipid metabolism in hepatocytes (50, 57). They were Sjp40 and Sjp90α. In previous experiments, the hepatic steatosis and insulin resistance of mice fed a high-fat diet were significantly improved by intraperitoneal injection of Sjp40 for 10 weeks (50). The results of the current study also revealed an increased expression of Hsp90α in mice fed a high-fat diet who were injected with Sjp40 (**Supplementary Figure S4**). This indicates that increased Hsp90α might be beneficial for fatty liver disease. It was initially hypothesized that increased expression of endogenous Hsp90α might be beneficial to hepatic metabolism. In the *in vitro* study, GGA induced Hsp90α in FL83B cells and improved steatosis. However, there was no increase in hepatic Hsp90α after the GGA treatment. Interestingly, an analysis of colocation immunofluorescence stain showed increasing Hsp90α in Kupffer cells rather than hepatocytes. Hsp90α was reported to increase M2 macrophages, which suppress inflammation (58, 59). Inflammation in the liver was reduced according to the current study. As expected, GGA showed encouraging efficacy in MAFLD. As experiments have shown, the NASH diet for mice treated with GGA for 12 weeks showed a remarkable reduction in steatosis and inflammation. Their insulin resistance and glucose intolerance were also improved. It seems that GGA may affect other pathways that are much more important than inducing Hsp90α.

Increased phosphorylated AMPK and ACC suggest that the drug enhanced fatty acid oxidation and reduced fatty acid synthesis. Thus, GGA may improve MAFLD through multiple pathways, including the regulation of energy metabolism and inhibition of inflammation. At first, to be known as an HSP inducer, GGA was found to induce HSPs other than Hsp90α. It was believed that GGA induced HSPs through HSF-1. GGA also induces many other HSPs, including Hsp70s, which are thought to be deficient in T2DM. It was reported that the expression of HSP72 is decreased in insulin resistance in T2DM. Insulin resistance and glucose metabolism were improved after restoring HSP72 by various methods, such as the transgenic overexpression of HSP72 in mice (60). We found Hsp70 decreased both in HFD animal models and in the hepatocyte steatosis experiments (**Supplementary Figures S4** and **S5**). It has also been reported that GGA reduces visceral fat and improves insulin resistance by inducing HSP72 in high-fat diet mice (22). Many other studies have shown that the improvement in liver fibrosis by GGA also depends on the induction of HSP70 (61). Takemasa Senoo’s study found that GGA improved carbon tetrachloride-induced liver fibrosis in mice. However, when GGA stimulated hepatic stellate cells (HSCs) *in vitro*, the expression of HSP70 in HSCs did not increase (62). Still, no such induction of Hsp70 was seen in our cell experiments (**Supplementary Figure S5**). In the mice model study, a greater than fourfold increase in HSP70 expression was observed in the CON-GGA group but not in the NASH-GGA group (**Supplementary Figure S6**).

GGA may have other mechanisms besides the induction of HSPs. As a terpene derivative, GGA is structurally similar to geranylgeranyl pyrophosphate (GGPP)/farnesylpyrophosphate (FPP) (https://www.chemicalbook.com/). GGPP/FPP is a reaction intermediate during the mevalonate pathway before the synthesis of terpenes or terpenoids. GGPP is synthesized by GGPP synthase from FPP. FFP/GGPP is involved in many signals in organisms. The liver X receptor (LXR)/farnesol receptor (FXR) are nuclear receptors involved in lipid metabolism. The synthesis and transport of intrahepatic triglycerides to the periphery are regulated by LXR. LXR also regulates the transport of cholesterol into the liver to reduce extrahepatic lipotoxicity. In the liver, the overexpression of LXR aggravates fatty liver. Antagonists of LXR attenuate steatosis, inflammation, and abnormal collagen deposition in mice with high-fat-diet-induced hepatic steatosis/steatohepatitis (63). In the liver, *de novo* lipogenesis (DNL) synthesizes fatty acids from acetyl-CoA. This process is regulated by SREBP1c and ChREBP. These two transcription factors regulate the expression of enzymes involved in the synthesis of fatty acids from acetyl-CoA. The inhibition of DNL has been identified as a therapeutic approach for steatohepatitis through the downregulation of SREBP1c. The suppression of specific enzymes in the fatty acid synthesis pathway is also a therapeutic target (64). Conversely, the activation of FXR reduces SREBP1c, thereby suppressing DNL (65). ACC is one of the essential enzymes in DNL regulated by SREBP1c. The increase in pACC in the GGA-treated group suggests a mechanism of DNL inhibition by GGA. Moreover, the activation of FXR inhibits the expression of NF-κB and reduces the release of proinflammatory cytokines (66). A synthetic FXR agonist was reported to improve high-fat-cholesterol diet-induced hepatic steatosis and insulin resistance (67). GGPP is the direct inhibitor of LXR by repressing the ATP-binding cassette protein A1 gene (68). Additionally, metabolites of FPP, including farnesol, also activate nuclear factors, particularly FXR (69, 70). Furthermore, LXR/FXR is regulated by FPP/GGPP (68, 71, 72).

Another important pathway GGPP/FPP is involved in the protein isoprenylation pathway. In organisms, hundreds of proteins undergo isoprenylation after translation. This modification anchors the protein to the cell membrane and mediates protein-protein interactions and signal transduction (73). Isoprenediyl for these proteins is obtained from GGPP/FPP. The most widely studied group of proteins requiring isoprenylation is the Ras superfamily, small GTPases, for example, Ras, Rho, Rab, Ran, and Arf (74). Some of these proteins are involved in the oxidative stress pathway. In the heart, the inhibition of isoprenylation of Rac1 may depress Rac1-mediated NADPH oxidase activation and ROS release (75). The inhibition of farnesylation has also been shown to benefit Alzheimer’s disease by reducing amyloid-β (76). It has been reported that GGA improves Alzheimer’s disease by inhibiting amyloid-β (77). Another study also reported that GGA blocked the function of GGPP by competitively inhibiting the mevalonate pathway and that this inhibition could be reversed by GGPP (78).

MAFLD is gaining attention for its increasing prevalence and hazards to health. However, noninvasive biomarkers and effective pharmacological treatments are still under investigation. This study presents a new biomarker, Hsp90α, whose expression and secretion are induced by inflammation. Hsp90α elevated both in MAFLD patients and the mouse model. Serum Hsp90α levels showed a linear relationship with the degree of hepatic steatosis and inflammation, which better reflected the activity of the disease. The predictive model based on Hsp90α combined with BMI, HbA1c, and ALT has good sensitivity and specificity for predicting MAFLD. We also demonstrated for the first time that teprenone improves MAFLD through multiple pathways, including reduced weight gain, increased glucose tolerance, improved insulin resistance, and even reversing hepatic steatosis and steatohepatitis. However, further research is needed to refine such models by recruiting more subjects and using more sensitive quantitative assessment methods. In addition, more studies on the mechanisms of GGA are needed.

## Conclusion

In the study, we found that serum Hsp90α was elevated in MAFLD, and a positive correlation between serum Hsp90α and the grade of activity of steatohepatitis was observed. The model using BMI, HbA1c, and ALT had a good value to predict MAFLD. The findings also revealed the effectiveness of GGA in the treatment of MAFLD.

## Data Availability Statement

The raw data supporting the conclusions of this article will be made available by the authors, without undue reservation.

## Ethics Statement

The studies involving human participants were reviewed and approved by the institutional review board of Sir Run Run Hospital, Nanjing Medical University. The patients/participants provided their written informed consent to participate in this study. The animal study was reviewed and approved by the Ethical Research Committee of Nanjing Medical University. Written informed consent was obtained from the owners for the participation of their animals in this study.

## Author Contributions

Conceptualization: YX, LuC, ZX, MJ, and YuL. Clinical research: YX, RH, RC, LQ, YaL, and YiZ. Experiments: YX, CL, ZX, MH, LiC, HC, YY, HW, YN, and YuZ. Supervision: LuC, ZX, MJ, and YuL. Writing, review, and editing: YX, YaL, MJ, and YuL. Funding acquisition: MJ, YuL, and YaL. All authors contributed to the article and approved the submitted version.

## Funding

This work was supported by the National Key R&D Program of China (2016YFC1305000, 2016YFC1305005); Innovative and entrepreneurial team of Jiangsu Province (2018); National Natural Science Foundation of China(81770778, 81971965); Science and Technology Plan of Jiangsu Province-Social Development (BE2017738); Key Medical Talents Project of Jiangsu Province (ZDRCA2016088); and University Science Research Project of Jiangsu Province (19KJB320013).

## Conflict of Interest

The authors declare that the research was conducted in the absence of any commercial or financial relationships that could be construed as a potential conflict of interest.

## Publisher’s Note

All claims expressed in this article are solely those of the authors and do not necessarily represent those of their affiliated organizations, or those of the publisher, the editors and the reviewers. Any product that may be evaluated in this article, or claim that may be made by its manufacturer, is not guaranteed or endorsed by the publisher.

## Abbreviations

MAFLD, metabolic-associated fatty liver disease; NAFLD, nonalcoholic fatty liver disease; T2DM, type 2 diabetes mellitus; CVD, cardiovascular disease; CKD, chronic kidney disease; NAFL, nonalcoholic fatty liver; NASH, nonalcoholic steatohepatitis; CK-18, cytokeratin 18; GGA, geranylgeranylacetone/teprenone; AUROC, area under the receiver operating characteristic curve; *ip*. GTT, intraperitoneal glucose tolerance test; *ip*. ITT, intraperitoneal insulin tolerance; AUC, area under the curve; FXR, farnesol receptor; DNL, *de novo* lipogenesis; BMI, body mass index; HbA1c, glycosylated hemoglobin; IGT, impaired glucose tolerant; SBP, systolic blood pressure; DBP, diastolic blood pressure; HTN, hypertension; ALT, alanine aminotransferase; AST, aspartate aminotransferase; TC, total cholesterol; TG, triglyceride; HTG, hypertriglyceridemia; HDL-C, high-density lipoprotein cholesterol; Hypo-HDL, hypo-HDL-cholesterolemia; LDL-C, low-density lipoprotein cholesterol; UA, uric acid; Hsp, heat shock protein; BUN, blood urea nitrogen; Cr, creatinine.
