# Supplementary material for: Predictive Modeling of MAFLD Based on Hsp90α and the Therapeutic Application of Teprenone in a Diet-Induced Mouse Model
Source: Front Endocrinol (Lausanne). 2021 Sep 30;12:743202. doi: 10.3389/fendo.2021.743202 (PMC8515197; doi:10.3389/fendo.2021.743202)
Supplement: Supplementary file 1 [file DataSheet_1.docx]

Supplementary Material

**The sequences of primers in RT-PCR are in Supplementary Table S4**

**Antibodies involved in Western blotting**: anti-Hsp90α (A13501, Abclonal), anti-AMPK (10929-2-AP, Proteintech), anti-pAMPK (2535, Cell Signaling Technology), anti-ACC (ab45174, Abcam), anti-pACC (ab68191, Abcam), β-actin (ab6276, Abcam). (HRP)-conjugated secondary antibodies (BL003A, Biosharp).

**Antibodies involved in Immunohistochemical staining**: anti-Hsp90 (GB12284, Servicebio) HRP-conjugated secondary antibody (GB23301, Servicebio) .

**Antibodies involved in immunofluorescence staining**: anti-Hsp90α (A0365, Abclonal), anti-F4/80 (GB11027, Servicebio) anti-Albumin (ab207327, Abcam). Cy5-conjugated secondary antibody (GB27303, Servicebio), HRP-conjugated secondary antibody (GB23303, Servicebio).

**ELISA Kits**: Human Hsp90α (CSB-E13462h, CUSABIO), Mouse Hsp90α (CSB-E08312m, CUSABIO), Mouse insulin (CSB-E05071m, CUSABIO), Mouse IL-6 (JL18212, Shanghai Jianglai Biotechnology), IL-1β, (JL46166, Shanghai Jianglai Biotechnology) and TNF-α (JL10484, Shanghai Jianglai Biotechnology).

**Supplementary tables**

Supplementary Table S1. Correlations between serum Hsp90α and relative factors

|  | ***r*** | ***P*** |
| --- | --- | --- |
| **Age** | 0.275 | **0.000***** |
| **Sex** | -0.247 | **0.001***** |
| **Smoking** | 0.198 | 0.060 |
| **MAFLD** | 0.455 | **0.000***** |
| **BMI** | 0.203 | **0.005***** |
| **HbA1c** | 0.480 | **0.000***** |
| **SBP** | 0.183 | 0.082 |
| **DBP** | 0.146 | 0.169 |
| **ALT** | 0.317 | **0.000***** |
| **AST** | 0.207 | **0.005***** |
| **TC** | 0.065 | 0.382 |
| **TG** | 0.345 | **0.000***** |
| **HDL-C** | -0.360 | **0.000***** |
| **LDL-C** | 0.051 | 0.487 |
| **UA** | 0.148 | 0.044 |

MAFLD, metabolic-associated fatty liver disease; BMI, body mass index; HbA1c, glycosylated haemoglobin; SBP, systolic blood pressure; DBP, diastolic blood pressure; ALT, alanine aminotransferase; AST, aspartate aminotransferase; TC, total cholesterol; TG, triglyceride; HDL-C, high-density lipoprotein cholesterol; LDL-C, low-density lipoprotein cholesterol; UA, uric acid, *r,*correlation coefficient*; P, P* value.

The significance was determined using the Spearman test. Bold values mean statistical significant. * *P＜*0.05 (FDR)

Supplementary Table S2. Multiple linear regression analysis between serum Hsp90α and relative factors

|  | B | S.E. | S.B | 95% CI | *P* Value | R^2^/Adjust R^2^ | D-W | VIF |
| --- | --- | --- | --- | --- | --- | --- | --- | --- |
|  |  |  |  |  |  | 0.315/0.275 | 1.534 |  |
| Age | 0.065 | 0.026 | 0.175 | -3.733-0.116 | **0.012*** |  |  | 1.195 |
| Sex | -0.415 | 0.633 | -0.049 | 0.015-0.835 | 0.513 |  |  | 1.428 |
| MAFLD | 1.793 | 0.789 | 0.209 | -1.665-3.350 | **0.024*** |  |  | 2.152 |
| BMI | -0.241 | 0.099 | -0.197 | 0.236-0.047 | **0.015*** |  |  | 1.644 |
| HbA1c | 0.568 | 0.146 | 0.294 | -0.436-0.856 | **0.000*** |  |  | 1.451 |
| ALT | -0.002 | 0.029 | -0.013 | 0.280-0.054 | 0.931 |  |  | 5.683 |
| AST | 0.068 | 0.052 | 0.186 | -0.059-0.170 | 0.195 |  |  | 5.197 |
| HDL-C | -0.247 | 0.777 | -0.025 | -0.035-1.287 | 0.751 |  |  | 1.578 |
| TG | 0.196 | 0.146 | 0.100 | -1.781-0.485 | 0.181 |  |  | 1.396 |
| UA | -0.003 | 0.004 | 0.049 | -0.011-0.006 | 0.551 |  |  | 1.690 |

MAFLD, Metabolic-associated fatty liver disease; BMI, body mass index; HbA1c, glycosylated hemoglobin; ALT, alanine aminotransferase; AST, aspartate aminotransferase; HDL-C, high-density lipoprotein cholesterol; TG, triglyceride; UA, uric acid. B, Coefficients; S.E, Standard error; S.B, Standardized coefficients; CI, Confidence interval; D-W, Durbin-Watson test;

VIF, Variance inflation factor.

Bold values mean statistical significant. **P*<0.05

Supplementary Table S3. Logistic regressions examining risk factors for MAFLD

|  | B | S.E. | Wald | OR | 95% CI | *P* Value | Nagelkerke R2 | VIF | Hosmer-Lemeshow χ² |
| --- | --- | --- | --- | --- | --- | --- | --- | --- | --- |
|  |  |  |  |  |  |  | 0.755 |  | 1.980 |
| Hsp90α | 0.219 | 0.109 | 4.016 | 1.244 | 1.005- 1.541 | **0.045*** |  | 1.315 |  |
| BMI | 0.414 | 0.119 | 12.223 | 1.513 | 1.200- 1.909 | **0.000*** |  | 1.293 |  |
| HbA1c | 1.649 | 0.626 | 6.933 | 5.204 | 1.525- 17.762 | **0.008*** |  | 1.154 |  |
| ALT | 0.068 | 0.034 | 3.976 | 1.071 | 1.001- 1.145 | **0.046*** |  | 1.238 |  |
| HDL-C | -1.772 | 0.733 | 5.852 | 0.170 | 0.040- 0.714 | **0.016*** |  | 1.294 |  |
| LDL-C | 0.917 | 0.438 | 4.382 | 2.503 | 1.060- 5.909 | **0.036*** |  | 1.249 |  |

Hsp, heat shock protein; BMI, body mass index; HbA1c, glycosylated hemoglobin; ALT, alanine aminotransferase; HDL-C, high-density lipoprotein cholesterol; LDL-C, low-density lipoprotein cholesterol; B, Coefficients; S.E, Standard error; WALD, Wald statistic; S.E., Standard error; VIF, Variance inflation factor; OR, Odds ratio; CI, Confidence interval.

Bold values mean statistical significant. **P*<0.05

Supplementary Table S4. Primer in RT-PCR

| **Gene** | **Forward 5’ -3’** | **Reverse 5’ -3’** |
| --- | --- | --- |
| Hsp90α | AATTGCCCAGTTAATGTCCTTGA | CGTCCGATGAATTGGAGATGAG |
| Hsp90β | CCTGGGAACCATTGCTAAGTCT | GCCCGATCATGGAGATGTCT |
| Hsp70 | TGGTGCAGTCCGACATGAAG | GCTGAGAGTCGTTGAAGTAGGC |
| IL-6 | TAGTCCTTCCTACCCCAATTTCC | TTGGTCCTTAGCCACTCCTTC |
| TNF-α | ATAGCTCCCAGAAAAGCAAGC | TTGGTCCTTAGCCACTCCTTC |
| IFN-γ | ATGAACGCTACACACTGCATC | CCATCCTTTTGCCAGTTCCTC |
| β-actin | GTGACGTTGACATCCGTAAAGA | GCCGGACTCATCGTACTCC |

**Supplementary Figure legends**

Supplementary Figure S1. Area under the receiver operating characteristic (AUROC) curves of the model for predicting MAFLD using Hsp90α.

AUROC = 0.77 (95% CI 0.702 - 0.837, P = 0.000), Cut-off 2.86 ng/mL, sensitivity 77.9%, specificity 66.7%.

Supplementary Figure S2. The average number of grams of feed consumed by each mouse per week in different groups.

Supplementary Figure S3. Comparison of serum Hsp90α between different groups

Control, Healthy subjects, n=72; MAFLD, MAFLD patients with normal glucose tolerance, n=41; T2DM, T2DM patients without MAFLD, n=39, MAFLD+IGT, MAFLD patients with impaired glucose tolerance, n=8, MAFLD+T2DM, MAFLD patients with T2DM, n=64. MAFLD, metabolic-associated fatty liver disease; T2DM, type 2 diabetes mellitus; IGT, impaired glucose tolerance. All points of data are presented in a Violin plot. The significance among groups was determined using the Kruskal-Wallis test. **P*<0.05, ***P*<0.01, ****P*<0.001.

Supplementary Figure S4. Expression of hepatic heat shock protein in a diet-induced obesity mice model.

(Male C57BL/6 mice aged four weeks were administrated with Sjp40 or saline for ten weeks after being fed a high-fat diet (45% kcal fat, 35% kcal carbohydrate, and 20% kcal protein; Research Diets Inc.) for three weeks). CON: normal diet group+saline, n=3; HFD, high-fat diet group+saline, n=4; HFD+P40, high-fat diet and injected with Sjp40 group, n=4. Data are presented as mean ± SEM. The significance among groups was determined using the two-way ANOVA with LSD’s multiple comparisons test. * *P* < 0.05

Supplementary Figure S5. Expression of heat shock protein in FL83b cells.

CON: control group; HF: steatosis group (200μM Oleic acid + 100 μM palmitic acid ); HF+GGA: steatosis + 1μM GGA group; (Equal volume of solvent was added to the control group). Data are presented as mean ± SEM. The significance among groups was determined using the two-way ANOVA with LSD’s multiple comparisons test. * P < 0.05

Supplementary Figure S6. Expression of hepatic heat shock protein.

[4-5-week-old male C57BL/6 mice were fed a normal diet (CON group)/Gubra Amylin NASH diet (NASH group) for 12 weeks and were then administrated GGA/NS once a day for 12 weeks. The whole experiment lasted 24 weeks. CON-NS: normal diet +NS, n=4; CON-GGA: normal diet+GGA, n=6; NASH- NS: Gubra Amylin NASH diet+NS, n=7; NASH-GGA: Gubra Amylin NASH diet+GGA, n=7 ]. Data are presented as mean ± SEM. The significance among groups was determined using the two-way ANOVA with LSD’s multiple comparisons test.**P*<0.05, ***P*<0.01, ****P*<0.001.
