# Supplementary figures and images for: Predictive Modeling of MAFLD Based on Hsp90α and the Therapeutic Application of Teprenone in a Diet-Induced Mouse Model
Source: Front Endocrinol (Lausanne). 2021 Sep 30;12:743202. doi: 10.3389/fendo.2021.743202 (PMC8515197; doi:10.3389/fendo.2021.743202)

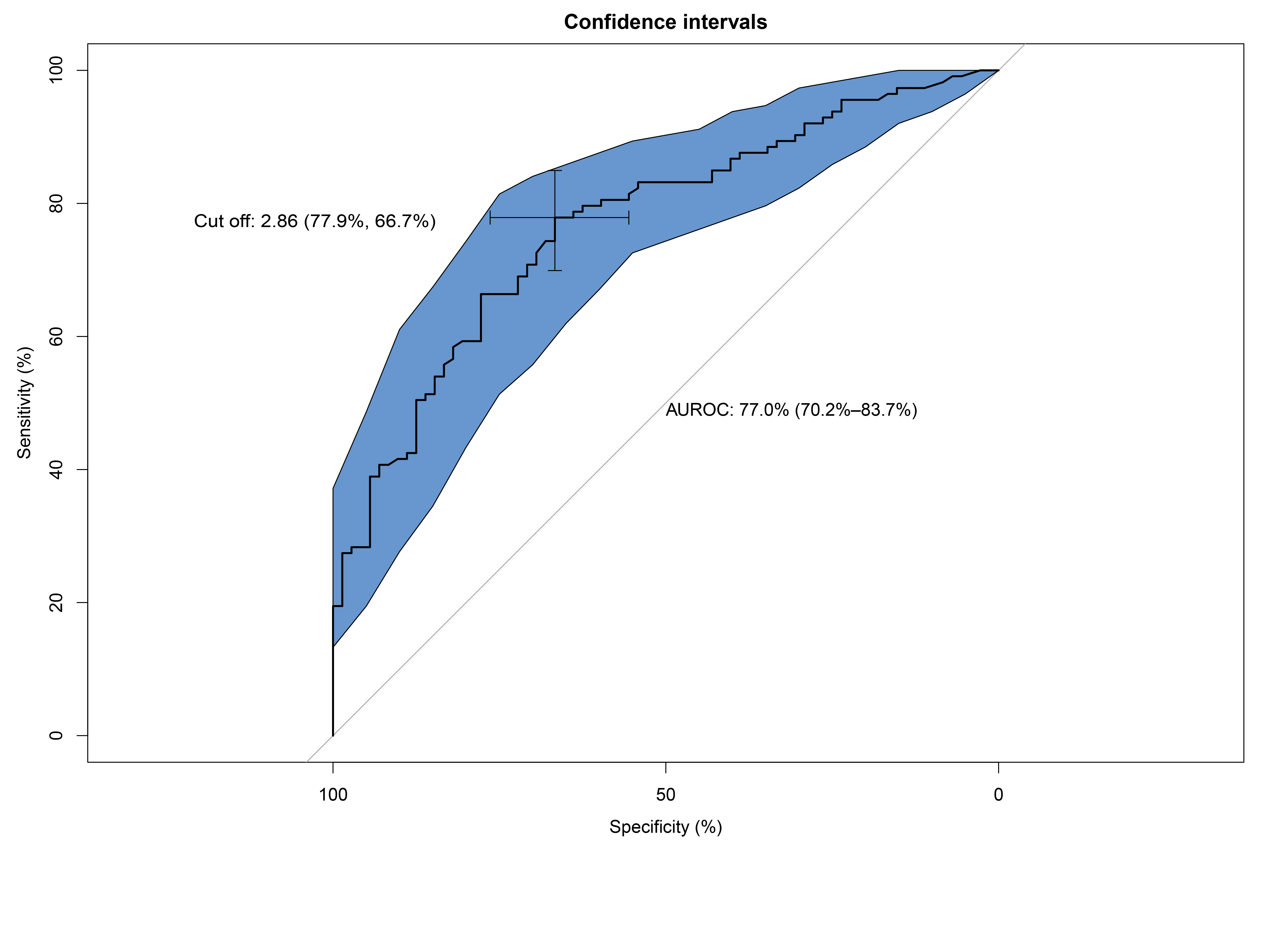

Supplement: Supplementary file 2 [file Image_1.tif]

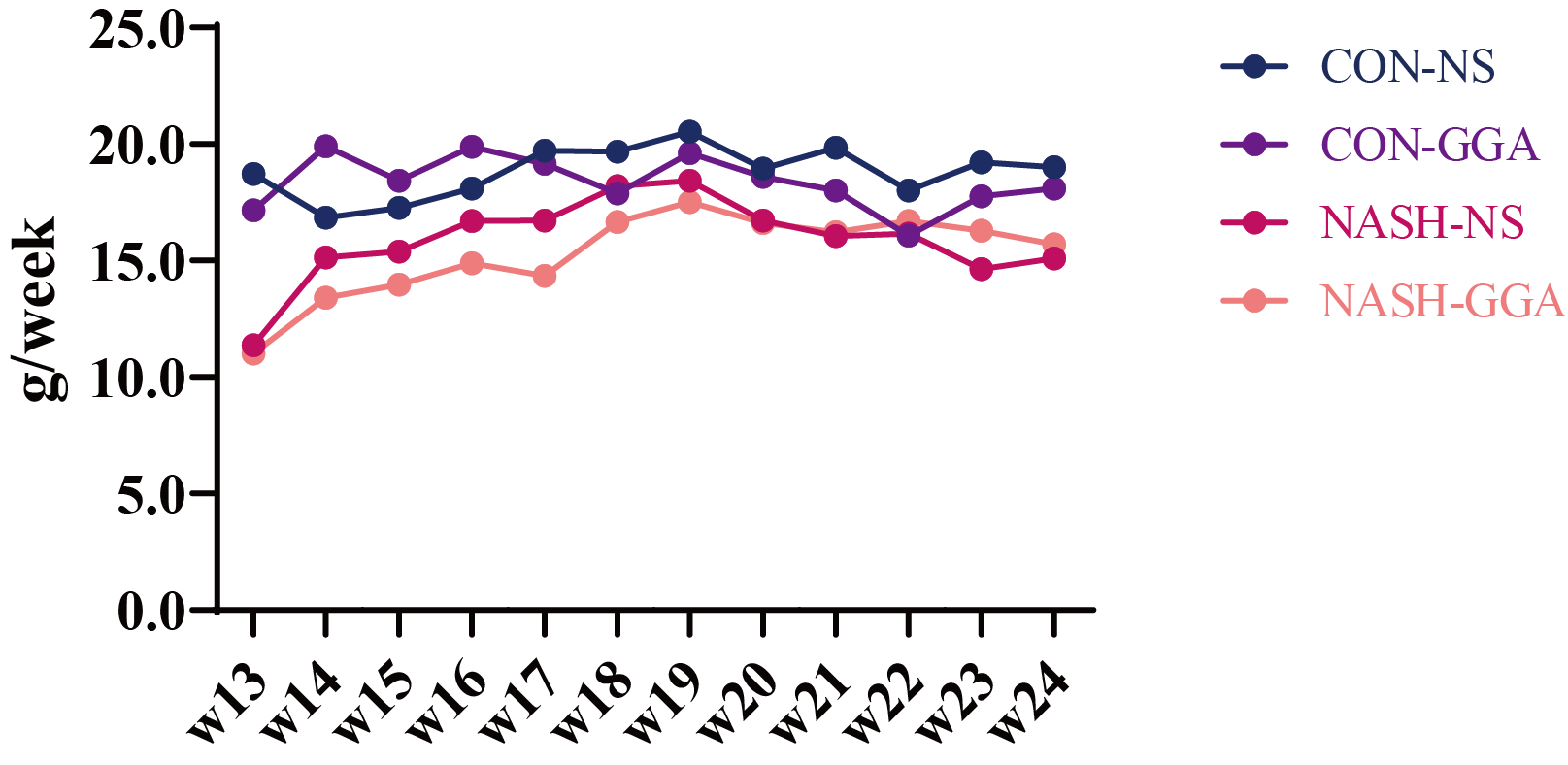

Supplement: Supplementary file 3 [file Image_2.tif]

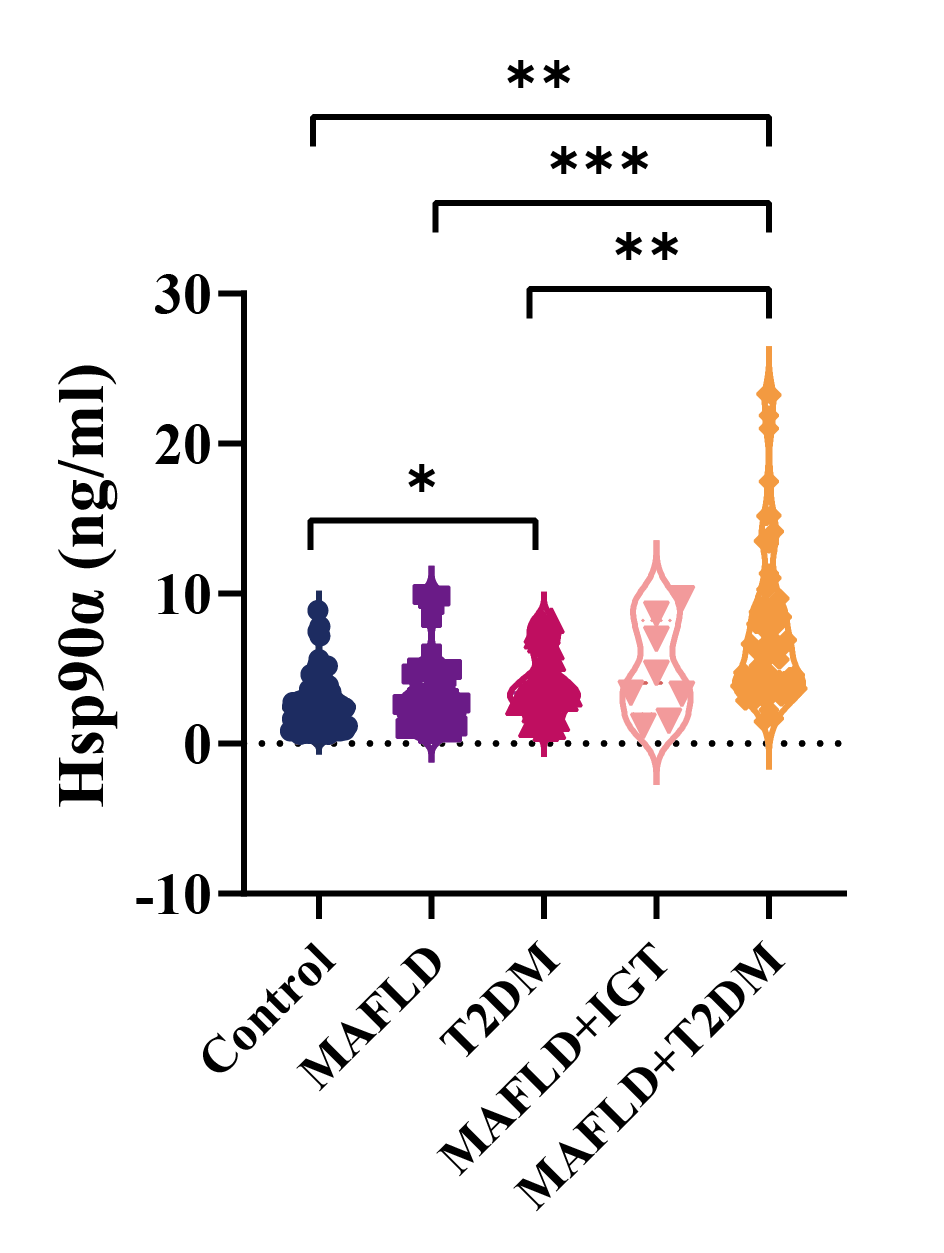

Supplement: Supplementary file 4 [file Image_3.tif]

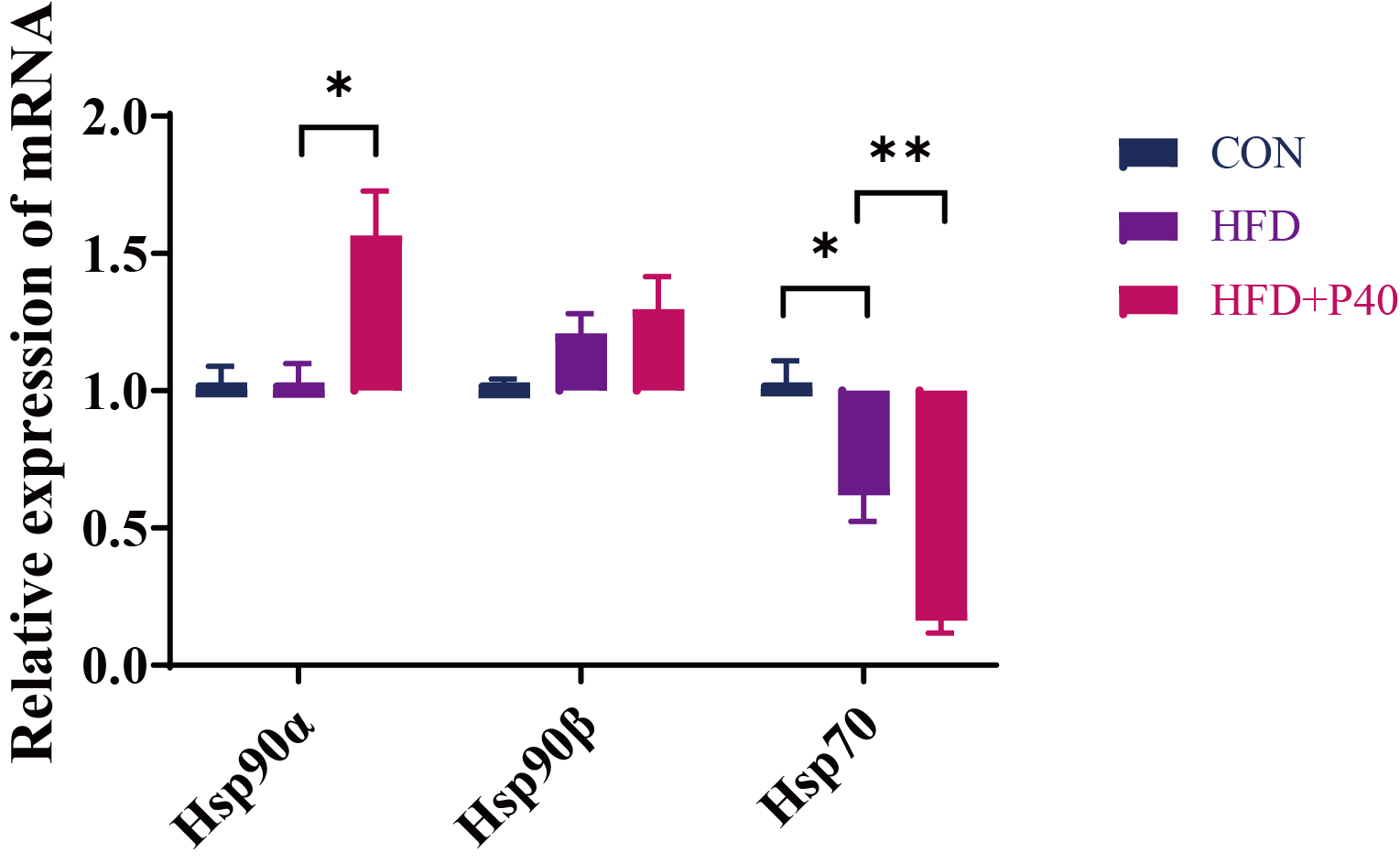

Supplement: Supplementary file 5 [file Image_4.tif]

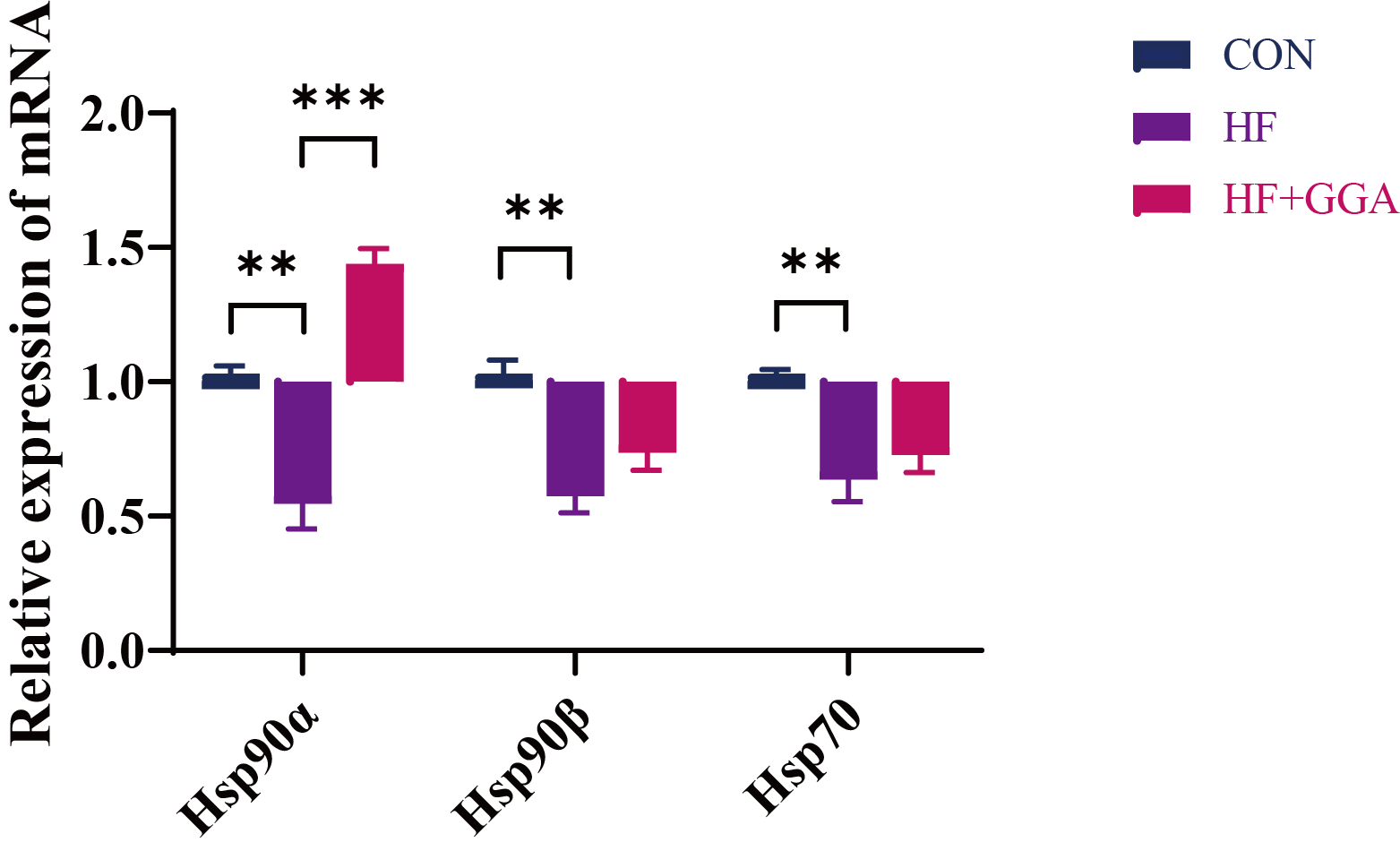

Supplement: Supplementary file 6 [file Image_5.tif]

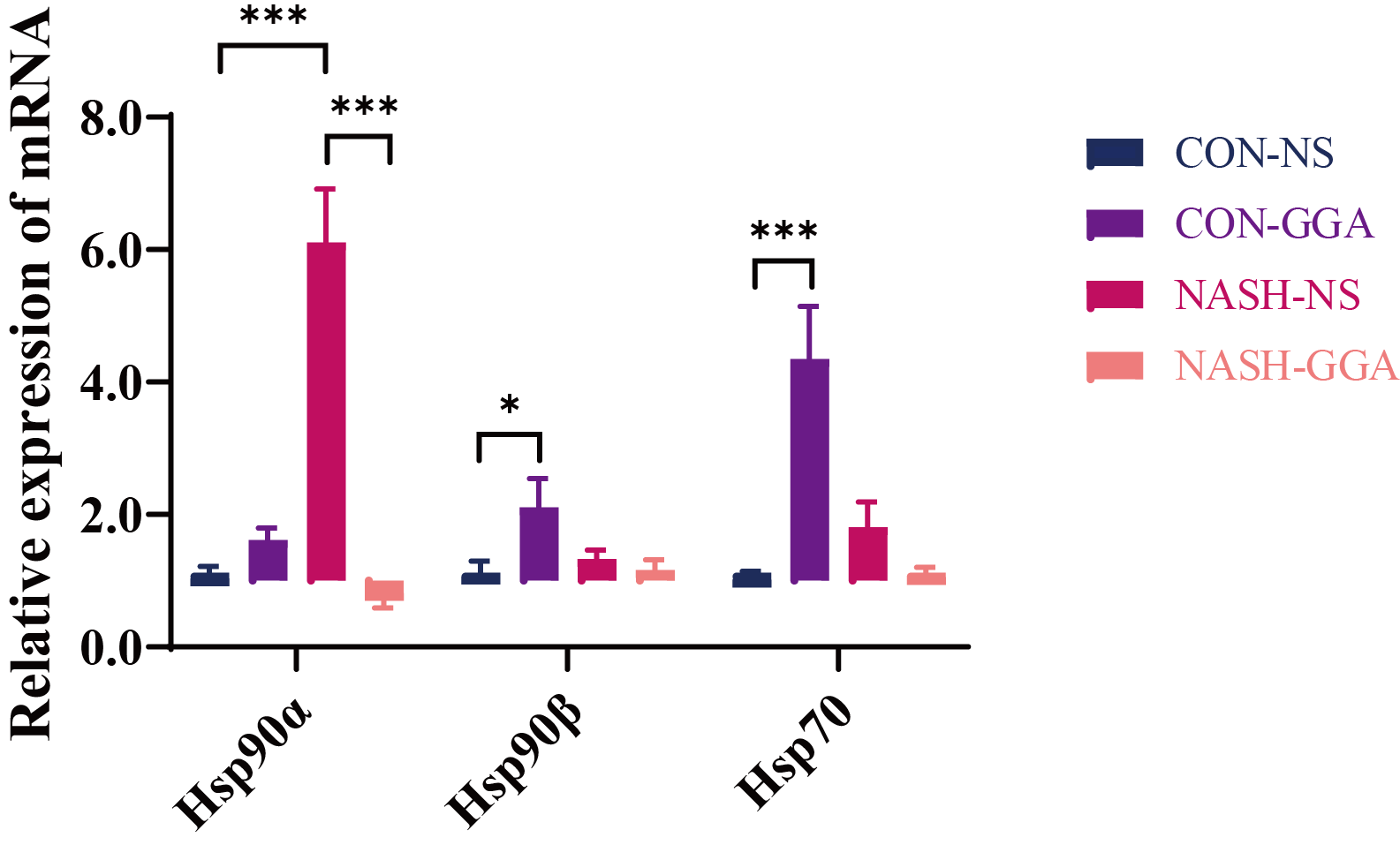

Supplement: Supplementary file 7 [file Image_6.tif]
